# A characteristic of the species composition of pathogenic fungi of the genus Fusarium in corn biocenoses of the Voronezh region

**DOI:** 10.18699/VJGB-22-71

**Published:** 2022-10

**Authors:** T.M. Kolomiets, M.I. M.I. Kiseleva, N.S. Zhemchuzhina, L.F. Pankratova, S.A. Elizarova

**Affiliations:** All-Russian Scientific Research Institute of a Phytopathology, Bolshie Vyazemy, Odintsovo district, Moscow region, Russia; All-Russian Scientific Research Institute of a Phytopathology, Bolshie Vyazemy, Odintsovo district, Moscow region, Russia; All-Russian Scientific Research Institute of a Phytopathology, Bolshie Vyazemy, Odintsovo district, Moscow region, Russia; All-Russian Scientific Research Institute of a Phytopathology, Bolshie Vyazemy, Odintsovo district, Moscow region, Russia; All-Russian Scientific Research Institute of a Phytopathology, Bolshie Vyazemy, Odintsovo district, Moscow region, Russia

**Keywords:** collections of microorganism, micromycetes, genetic diversity, corn, plant pathogens, Fusarium, коллекции микроорганизмов, микромицеты, генетическое разнообразие, кукуруза, фитопатогены, Fusarium

## Abstract

Corn is one of the main crops of modern world agriculture. It ranks f irst in terms of gross grain harvests and second in terms of acreage, ceding only to the main grain crop of the globe, wheat. The problem of increasing the production of grain and green mass of corn remains one of the urgent tasks of agricultural production. High potential yields very often remain untapped due to diseases, direct losses from which are estimated at 20–50 %. The purpose of this work was to study the species composition of micromycetes on corn collected in different phases of vegetation in May-July 2020 in the Voronezh region, to identify phytopathogenic genus Fusarium fungi, to study pathogenic and phytotoxic strains of the fungi to replenish the collection of the All-Russian Scientific Research Institute of a Phytopathology. Preservation of infectious material of fungi from the genus Fusarium is of no small importance for phytopathological, immunological, breeding, genetic and toxicological studies. As a result of the mycological studies carried out, a lot of fungi isolates from the genera Fusarium, Aspergillus, Cladosporium, Curvularia, Penicillium, Rhizopus, Periconia, Pythium, Trichothecium, etc., isolated from the affected roots, stems and ears of corn in the Voronezh region in 2020 were identif ied. Fungi isolates from seven taxonomic groups: Fusarium fujikuroi Nirenberg (F. moniliforme, F. verticillioides), Fusarium oxysporum Schltdl., Fusarium culmorum (Wm.G. Sm.) Sacc., Fusarium graminearum Schwabe, Fusarium heterosporum Nees & T. Nees (F. lolii ), Fusarium roseum Link (F. sambucinum), Fusarium sporotrichioides Sherb. were tested for pathogenicity and phytotoxicity on seedlings of plant-testers. It has been shown that pathogenic and phytotoxic activity in fungi varies signif icantly between Fusarium species and within the same species. The greatest danger to corn is represented by the species F. sporotrichioides, F. graminearum, F. culmorum, F. fujikuroi, F. oxysporum, F. heterosporum, which have a high intensity of phytotoxic activity associated with the fact that they contribute to the synthesis and accumulation of dangerous toxins in plant tissues. As a result of the conducted studies, 55 strains of fungi from the genus Fusarium belonging to seven species were selected. The isolates, stable in morphological and cultural characteristics and studied for pathogenicity and toxicity, were placed for long-term storage in the Russian State Collection of Plant Pathogenic Microorganisms and Cultivars for Identif ication of Phytopathogenic Microbial Strains at the All-Russian Scientif ic Research Institute of a Phytopathology.

## Abstract (ru)

Кукуруза относится к основным культурам современного мирового земледелия. Она стоит на первом месте по валовым сборам зерна и на втором – по посевным площадям, уступая лишь основной хлебной культуре земного шара – пшенице. Одна из актуальных задач аграрного производства – проблема увеличения валового сбора зерна и зеленой массы кукурузы. Высокая потенциальная урожайность очень часто остается нереализованной вследствие развития болезней, прямые потери от которых оцениваются в 20–50 %. Цель настоящей работы – изучение видового состава микромицетов на растениях кукурузы, собранных в разные фазы вегетации в мае-июле 2020 г. в Воронежской области, идентификация фитопатогенных грибов из рода Fusarium, выявление патогенных и фитотоксичных штаммов грибов рода Fusarium для пополнения коллекции Всероссийского научно-исследовательского института фитопатологии. Сохранение инфекционного материала грибов из рода Fusarium имеет немаловажное значение для фитопатологических, иммунологических, селекционных, генетических и токсикологических исследований. В результате проведенного микологического анализа обнаружено большое количество изолятов грибов из родов Fusarium, Aspergillus, Cladosporium, Curvularia, Penicillium, Rhizopus, Periconia, Pythium, Trichothecium и др., выделенных из пораженных корней, стеблей и початков кукурузы в Воронежской области в 2020 г. Изоляты грибов из семи таксономических групп: Fusarium fujikuroi Nirenberg (F. moniliforme, F. verticillioides), Fusarium oxysporum Schltdl., Fusarium culmorum (Wm.G. Sm.) Sacc., Fusarium graminearum Schwabe, Fusarium heterosporum Nees & T. Nees (F. lolii ), Fusarium roseum Link (F. sambucinum), Fusarium sporotrichioides Sherb., были проверены по патогенности и фитотоксичности на всходах растений-тестеров. По- казано, что патогенная и фитотоксичная активность у грибов как между видами Fusarium, так и в пределах од- ного вида существенно различается. Наибольшую опасность для кукурузы представляют виды F. sporotrichioides, F. graminearum, F. culmorum, F. fujikuroi, F. oxysporum, F. heterosporum, обладающие высокой интенсивностью про- явления фитотоксической активности, связанной с тем, что они способствуют синтезу и накоплению опасных токсинов в тканях растений. В результате проведенных исследований отобрано 55 штаммов грибов из рода Fusarium, относящихся к семи видам. Стабильные по морфолого-культуральным признакам и изученные по па- тогенности и фитотоксичности изоляты помещены на длительное хранение в Государственную коллекцию фи- топатогенных микроорганизмов и сортов растений-идентификаторов патогенных штаммов микроорганизмов, созданную на базе Всероссийского научно-исследовательского института фитопатологии.

## Introduction

Along with the fact that sugar corn (lat. Zéa máys L. ssp. mays) is the only cultural representative of the genus Corn (Zea) of the Cereal family (Poaceae) and the oldest bread plant in the world, it remains one of the most popular in the realities of modern agriculture (Sotchenko, 2005). Corn ranks first in terms of gross grain harvests and second in terms of acreage, second only to the main grain crop of the globe – wheat. The USA (about half of the world harvest), China, Brazil, Mexico, France, Argentina, India, Indonesia, Italy and Romania are the largest producers of corn (Babich, 1986; Berezkin, Malko, 1998; Elmore, Abendroth, 2008). Corn is cultivated mainly in the southern regions of Russia (Suprunov, 2009).

Due to the high yield and useful qualities of corn, its importance for versatile human use can hardly be overestimated. More than 20 % of corn grain is used for food purposes in the countries of the world, 15–20 % – for technical purposes, and about two thirds – for livestock feed (Sotchenko, 2009).

As a food crop, corn ranks third in the world in terms of acreage giving way only to wheat and rice. And in terms of grain yield, it has a leading position. Corn grain contains 65–70 % carbohydrates, 9–12 % protein, 4–8 % vegetable oil (up to 40 % in the embryo) and only about 2 % fiber. Corn grain contains vitamins A, B1, B2, B6, E, C, D, F, essential amino acids, mineral salts and trace elements. Corn grain is used for food and medical purposes (Sotchenko, 2002).

In the modern world, fodder corn yields large harvests and highly nutritious feed which makes it crucial in the development of husbandry. Corn plays the main role in the feed balance because of its caloric characteristics and the possibility of using both corn grains and its green mass – silage (https// universityagro.ru/растениеводство/кукуруза/; Ivashchenko, Sotchenko, 2006; Sotchenko, Gorbacheva, 2011).

Corn is also of great importance for industry. Corn oil is a raw material for the production of expensive paints, soaps and rubber substitutes. Corn starch is used for dressing fabrics and leather, increasing the density and smoothness of paper, in the production of viscose fiber, explosives, dextrin glue. Construction and packaging materials, paper, soil improving additives, explosives are obtained from stems and other vegetative parts of plants. Furfural, a raw material for the production of plastics, nylon and other synthetic substances, is isolated from the stalks of corn cobs (https//universityagro. ru/растениеводство/кукуруза/).

The problem of increasing corn grain production remains one of the urgent tasks of agricultural production (Sotchenko, 2005). In Russia, high potential yield of corn often remains unrealized due to the development of diseases, among which the main role belongs to micromycetes from the genera Fusarium, Bipolaris, Alternaria, etc. Direct grain losses from Fusarium root and ear rot in Corn at 20–50 % (Ivashchenko, 2007, 2012).

Fusarium root and ear rot are widespread corn diseases, especially in areas with high humidity. Up to 50–60 % of corn crops are affected. A large group of maize diseases are fungi from the genus Fusarium: Fusarium acuminatum Ellis & Everh, F. culmorum (W.G. Sm.) Sacc., F. equiseti (Corda) Sacc., F. gibbosum Appel. et Wollenw., F. graminearum Schwabe, F. heterosporum Nees & T. Nees, F. oxysporum f. conglutinans (Wollenw.) W.C. Snyder & H.N. Hansen, F. oxysporum f. cucumerinum Berk. & Broome, F. poae (Peck) Wollenw., F. roseum Link, F. solani (Mart.) Sacc., F. sporotrichioides Sherb. and others (Ali et al., 2005; Eller et al., 2008).

Fusarium ear rot in Corn caused by the hemibiotrophic fungus Fusarium verticillioides (Sacc.) Nirenberg (syn. Fusarium moniliforme J. Sheld., marsupial stage – Gibberella fujikuroi) leads to a decrease in yield and deterioration of its quality (Miller et al., 2007; Murillo-Williams, Munkvold, 2008; Mesterhasy, Lemmens, Reid, 2012). The fungus produces fumonisins when storing cobs in conditions of high humidity and insufficient aeration. These toxins are carcinogenic to humans and animals (Clements, White, 2004; Robertson-Hoyt et al., 2007).

As for the species that cause root rot, low temperature during seed germination, increased humidity and soil acidity increase the development of the disease (Suprunov, 2009). At the same time, a weak pink or white fungus bloom forms on the surface of the germinating grain. Soon after the corn plants come to the surface, the sprout turns brown and dies. If the sprout survives, then it has a poorly developed root system, plants are delayed in growth, leaves dry up, often lie down (Ivashchenko et al., 2006).

Since the pathogens of Fusarium root and ear rot reside in the soil and grain, the question of studying the range of the most pathogenic micromycetes, including those from the genus Fusarium, is relevant for the development of environmentally friendly methods of combating them, including the creation of disease-resistant varieties and hybrids of corn (Hooker, 1967; Ivashchenko, 2009a; Ivashchenko, Matveeva, 2010). Particular attention is paid to the creation of infectious backgrounds, where measures are carried out to assess and select resistant forms (Ivashchenko, 2007, 2009b). The preparations of compositions of infectious backgrounds, which includes the study of the species composition of corn micromycetes, the identification of the most pathogenic isolates of fungi of the genus Fusarium and the creation of conditions for their long-term storage without loss of pathogenic properties, are also of importance

Mycological analysis of maize samples and analysis of the scientific literature related to the issue under development indicates that monitoring the species composition of fungi on the cultivated crop is currently very relevant both for taking urgent preventive and health measures and for developing a strategy to prevent negative consequences from the development of diseases. Researches aimed at studying the species composition of micromycetes that cause Fusarium root and ear rot ultimately determine the possibility of obtaining environmentally friendly and stable corn crops.

Preservation of infectious material of fungi from the genus Fusarium is important for phytopathological, immunological, breeding, genetic and toxicological studies (Dubovoy et al., 2016; Kolomiets et al., 2018; Kolomiets, Zhemchuzhina, 2018). The State Collection of Phytopathogenic Microorganisms and Plant Varieties, identifiers of pathogenic strains of microorganisms of the All-Russian Scientific Research Institute of Phytopathology (GKFM VNIIF), was created to solve the tasks set in accordance with Federal Law No. 7-F3 of 10.01.02 (ed. of 24.11.2014, with amendments dated 29.12.2014) “On Environmental Protection”, Decree of the Government of the Russian Federation No. 725-47 dated 24.06.1996 “On Measures for the Conservation and Rational Use of Collections of Microorganisms, Cultivated Cells of Higher Plants, Transplanted Somatic Cells of Vertebrates”, as well as taking into account the provisions of the Convention on Biological Diversity (1992) and the recommendations of the European Organization for Economic and Social Development (GENERAL GUIDELINES FOR ALL BRCS, 2006; GUIDANCE FOR THE OPERATION OF BIOLOGICAL RESOURCE CENTERS (Part 2: Micro-Organization Domain), 2006; OECD Best Practice Guidelines for BRCs 2007). It is a State Depository of phytopathogenic microorganisms

As for the creation of a collection of fungi from the genus Fusarium, its main tasks were not only to preserve the viability and genetic stability of strains of these fungi according to morphological and cultural characteristics for a long time, but also to replenish the fund with new species with a different spectrum of pathogenicity and phytotoxicity properties, as well as to expand the range of geographical areas for collecting affected maize samples (Gagkaeva, Levitin, 2005; Gagkaeva et al., 2008). To fulfill these tasks, samples of infected plants received annually from various regions of the country are subjected to mycological studies, and based on the data of the analysis of the material, the most pathogenic and phytotoxic samples are selected for the collection.

The purpose of this work was to study the species composition of micromycetes on corn plants collected in different phases of vegetation in May–July 2020 in the Voronezh region, to identify pathogenic and phytotoxic strains of fungi of the genus Fusarium to replenish the collection of the ARSRIP.

## Materials and methods

Maize plants with various signs of fungal infections on leaves, stems and roots served as the material for research. Samples of zoned varieties of corn (Ajaks, Donskaya visokoroslaya, Zernogradsky) were collected in different phases of vegetation: the formation of 5–6 leaves – f.2, according to the classification of phenological development according to the system of ВВСН, tubing, or the formation of 8–10 leaves – f.32, filling – milk ripeness – f.75 (Large, 1954; Lancashire et al., 1991). Research was carried out using the equipment of the Collective Use Center of the Russian State Collection of Plant Pathogenic Microorganisms and Cultivars for Identification of Phytopathogenic Microbial Strains at the All-Russian Scientific Research Institute of a Рhytopathology (http://www. vniif.ru/vniif/page/ckp-gkmf/1373).

The phytosanitary condition of the samples was assessed according to methods generally accepted in phytopathology (Gerlach, Nirenberg, 1982; Leslie, Summerell, 2006; Dictionary..., 2008; Watanabe, 2010). Fungal species were determined by the morphology of spores under a microscope ×400 (Bilai, 1977; Bilai, Ellanskaya, 1982; Gagkaeva et al., 2008).

The isolation of hemibiotrophic and saprophytic micromycetes from the affected plants was carried out using potato- glucose and potato-carrot agar-agars. Fungi from plant samples were isolated according to the standard method (Bilai, 1977; Bilai, Ellanskaya, 1982). The diseased plants of each sample were washed with tap water and then were cut into fragments 5–10 mm in size, sterilized in 50 % alcohol for 20–30 seconds and, under aseptic conditions, were laid out on the surface of 2 % potato-glucose agar-agar in Petri dishes (4–6 fragments in each). Each sample was represented by at least 150–200 fragments of the affected tissue. Petri dishes were placed in a thermostat with a temperature of 22–24 °С. The development of fungi was monitored daily. As the colonies of fungi grew, a piece of mycelium was sifted onto the nutrient medium in the center of the Petri dish. Cultures of fungi were viewed under a microscope. Fungal species were identified by the main morphological features of colonies and spores: by growth rate, mycelium color and structure, pigmentation; by shape, size of apical and basal cells of macroconidia, by the presence of microconidia. An average microscopy index of 300 conidia was taken to estimate the size of macroconidia.

Determinants were used as reference literature when determining the species of the fungus (Gerlach, Nirenberg, 1982; Bilai, Kurbatskaya, 1990; Leslie, Summerell, 2006; Dictionary..., 2008; Watanabe, 2010). The current taxonomic status of the selected Fusarium species was clarified at http://www. indexfungorum.org.

The frequency of occurrence of original Fusarium species in samples of affected plants as a percentage was determined by the formula

**Formula. 1.**
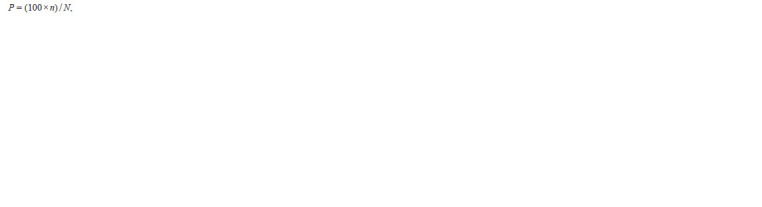
Formula 1.

where P is the frequency of occurrence of the species in the population (in %); N is the total number of isolates of fungi of the genus Fusarium in the sample; n is the number of isolates of a certain type of Fusarium in the sample.

Isolates of fungi isolated from the affected corn samples were placed for storage in the laboratory of the Russian State Collection of Plant Pathogenic Microorganisms and Cultivars for Identification of Phytopathogenic Microbial Strains at the All-Russian Scientific Research Institute of a Phytopathology. Isolates have been stored in refrigerators at a temperature of 7–10 °С in biological test tubes on slants of nutrient medium – potato-glucose agar-agar (Bilai, Ellanskaya, 1982).

Pathogenic and toxic properties of strains were studied using the method of bioassay on seeds. The pathogenicity of spore suspensions and phytotoxicity of filtrates of culture fluids (FCF) of fungi were tested on wheat seeds (cv. Mironovskaya 808). The degree of pathogenicity and toxicity of strains was judged by the effect of suspensions of conidia and FCF on seed germination, the development of germ and primary roots of wheat, but the main indicator was the length of the roots.

The degree of pathogenicity and toxicity was determined on the 5th day from the beginning of seed germination. If the length of seedlings and roots (in mm) in the experimental version was 0–30 % of the length of the control, then this indicated a strong pathogenic (P) and strong toxic (T) activity of the fungus; 31–50 % – moderate pathogenicity (MP) and moderate toxicity (MT); 51–70 % – weak pathogenicity (WP) and weak toxicity (WT); 71–100 % – non-pathogenic (NP) and non-toxic (NT) properties of isolates. The length of the sprouts and primary roots of seeds germinated in water was considered as a control and was taken as 100 % (Parfenova, Alekseeva, 1995).

## Results and discussion

Mycological studies of the analyzed maize plants collected in different phases of vegetation (formation of 5–6 leaves, tube formation, milk ripeness) showed the presence of micromycetes on them, related to both phytopathogens and saprotrophs. In total, more than 30 species of micromycetes were isolated and identified from corn samples

Saprotrophic species of fungi from the genera Aspergillus, Cladosporium, Curvularia, Penicillium, Rhizopus, Periconia, Pythium, Trichothecium, etc. prevailed on the tissues of the roots and basal areas of corn stalks (Table 1). Heterotrophic species of fungi were more often found on the leaves of the samples. Almost half (1600 units) of the fungi isolates identified from the leaves and roots belonged to the genera Alternaria, Bipolaris, Exserohilum and Fusarium. It should be noted that the frequency of occurrence of fungi Alternaria spp. depended on the phenological phase of corn plants. So, in the phase of formation of 5–6 leaves, fungi of this genus were significantly more often isolated from the tissues of corn roots, in the phase of milk ripeness – from the leaves.

**Table 1.**
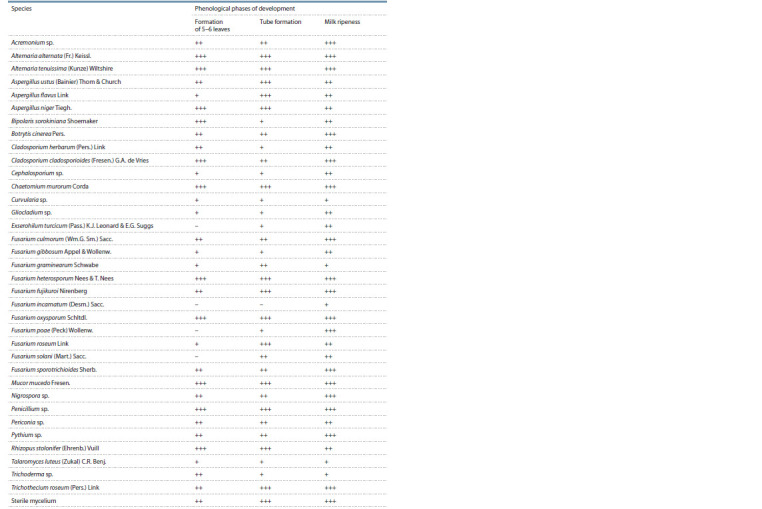
Micromycetes found on corn crops in the Voronezh region in 2020 Notе. ”+“ – from 1 to 10 fungus isolates; ”++“ – from 11 to 20 fungus isolates; ”+++“ – above 20 fungus isolates.

Symptomatic analysis of maize samples revealed signs of infection with the pathogen Exserohilum turcicum (Pass.) K.J. Leonard & E.G. Suggs (=Setosphaeria turcica). On the leaves of the corn of the lower tier, large spots were noted, gray in the center and with darker edges with a sooty coating. Samples with such signs were found in the phase of tube formation and milk ripeness, the intensity of their lesion was low and ranged from 1 to 20 % of the leaf area of the lower tier.

Isolates of Bipolaris sorokiniana Shoemaker (Cochliobolus sativus) were mainly found on the roots and basal part of corn stalks during the 5–6 leaf formation phase. The fungus was not identified on the leaves during this and later phenophases

The manifestation of diseases caused by fungi from the genus Fusarium had similar symptoms. As a rule, brown or yellow areas were noted on the leaves, stems, basal neck and roots of corn, often with signs of maceration or rottenness. The study of samples of the affected tissues of maize plants in culture allowed to isolate more than 900 isolates of the genus Fusarium into a culture and identify by morphological characteristics (colony growth rate, mycelium color and structure), the presence, shape and size of macroconidia and microconidia (if present) the following 11 species of this genus: F. culmorum, F. gibbosum, F. graminearum, F. heterosporum, F. fujikuroi, F. incarnatum, F. oxysporum, F. poae, F. roseum, F. sporotrichioides, F. solani (Table 2). In some cases, more than one or two micromycetes from the genus Fusarium were isolated from one sample of affected corn tissue. This was especially often noted when F. oxysporum was isolated into culture, which, as a rule, was accompanied by the species F. roseum, F. poae, F. solani, etc.

**Table 2.**
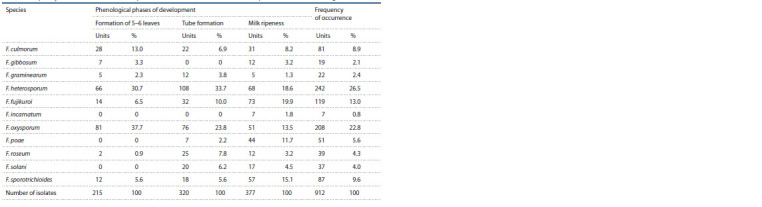
Frequency of occurrence of Fusarium species detected on the affected maize samples from the Voronezh region in 2020

The frequency of occurrence of fungi from the genus Fusarium was ambiguous and varied markedly depending on the phase of the growing season of corn and possibly the prevailing weather conditions of the season. The species F. heterosporum and F. oxysporum were most often found in the complex of micromycetes from the genus Fusarium on corn crops in the Voronezh region. The total share of these two species was half of all identified isolates belonging to other species of this genus. Nevertheless, fluctuations in the frequency of occurrence of these types of fungi were observed in all phenological phases of corn development. When assessing the frequency of occurrence of species from the genus Fusarium in the phase of milk ripeness, it was noticed that the proportion of F. heterosporum and F. oxysporum isolates decreased by 1.5–2 times (see Table 2). It should also be pointed out that F. heterosporum isolates were more often isolated from affected corn roots, and F. oxysporum, from stems.

In mycological studies of corn tissues, isolates of F. fujikuroi were found in all variants of the experiment. The frequency of occurrence of the fungus gradually changed from low (6.5 %) in the phase of formation of 5–6 leaves to high (19.4 %) in the phase of milk ripeness. Probably, over time, more favorable conditions for the accumulation of F. fujikuroi in the soil and on maize plants had been created. A similar pattern was observed for the species F. poae and F. sporotrichioides, the frequency of occurrence of which varied significantly from the phase of formation of 5–6 leaves to the phase of milk ripeness, respectively, from low (0 and 5.6 %) to high (11.7 and 15.1 %).

As for F. culmorum, there were no significant fluctuations in the frequency of occurrence of the fungus on maize samples in different phenological phases. This indicates a sufficiently high viability of the micromycete, which occupies a certain niche in the Fusarium spp. pathocomplex. As a rule, macroconidia of the fungus were detected on the affected samples from the roots and leaves of the lower tier.

Species of F. roseum, F. solani, F. graminearum, F. gibbosum, F. incarnatum in the pathogenic complex of the Fusarium fungi on corn were quite rare. Basically, isolates of these micromycetes were determined on the affected roots and the root zone of the stems. It is possible that either these types of fungi do not play a significant role in the pathogenesis of corn, or there were no conditions for their development.

As a result of mycological studies, biological material was obtained represented by a large number of fungal isolates: 11 species from the genus Fusarium. Of them, 55 isolates of fungi from 7 taxonomic groups (F. fujikuroi, F. oxysporum, F. culmorum, F. graminearum, F. heterosporum, F. roseum, F. sporotrichioides) were tested for pathogenicity and phytotoxicity on seedlings of testers

Table 3 shows the results of assessing the effect of metabolites of spore suspensions and filtrates of culture fluids of fungal isolates of the most pathogenic and phytotoxic species of the genus Fusarium on the development of wheat seedlings of cv. Mironovskaya 808 (seed germination, length of the sprout and roots). It was shown that isolates of fungi from the genus Fusarium represented by different species had a wide intraspecific diversity in the studied characteristics. Within the same species, there were strains of the fungus belonging to different categories – from pathogenic/toxic to non-pathogenic/non-toxic (see the Figure).

**Table 3.**
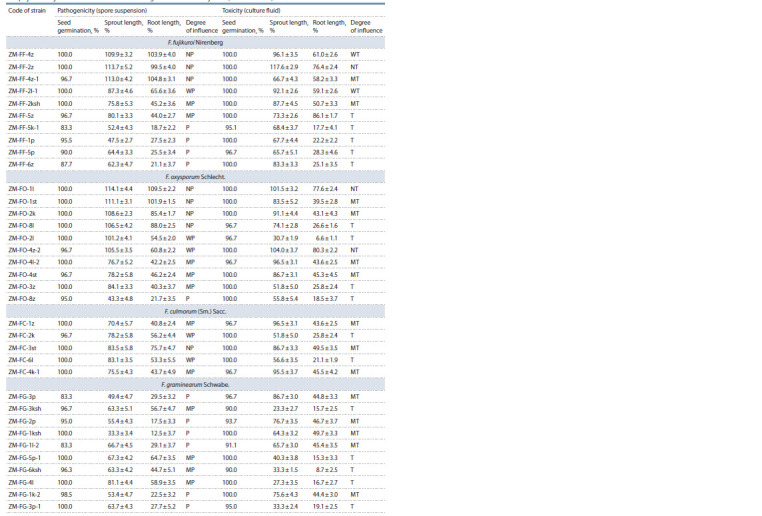
Characteristics of strains of fungi of seven species from the genus Fusarium by pathogenicity of spore suspensions and phytotoxicity of culture fluid on wheat seedlings of cv. Mironovskaya 808 (in % of control) Notе. NP/NT is non-pathogenic/non-toxic; WP/WT – weakly pathogenic/weakly toxic; MP/MT – moderately pathogenic/moderately toxic; P/T – pathogenic/toxic.

**Table 3end.**
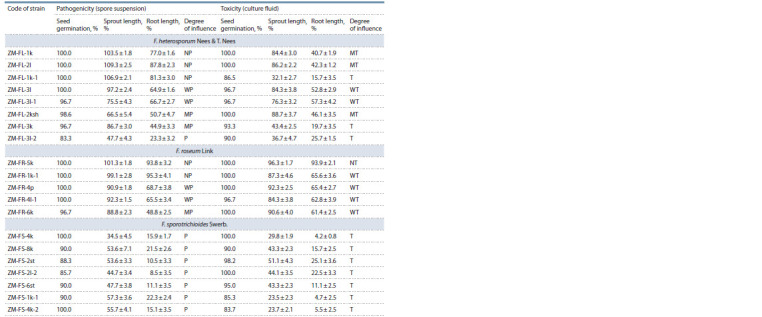
Table 3 (end)

**Fig. 1.**
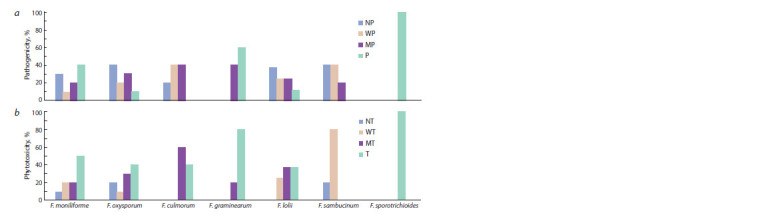
Distribution of fungal species from the genus Fusarium by pathogenicity (a) and phytotoxicity (b), %.

The species F. sporotrichioides and F. graminearum showed high phytotoxic and pathogenic properties. Culture fluid filtrates and spore suspensions of the isolates of these species almost completely suppressed the development of seedlings of plants of the tester variety

The species of fungi F. culmorum, F. fujikuroi, F. oxysporum, F. heterosporum had stronger phytotoxic properties than pathogenic, showing a moderately toxic and toxic reaction to the seedlings of the tester variety. The species F. roseum was characterized by weak pathogenicity and weak phytotoxicity.

## Conclusion

As a result of mycological analysis of the composition of micromycetes on affected maize plants in different phenological phases of plant development, more than 30 species of micromycetes were identified. Saprotrophic species of fungi from the genera Aspergillus, Cladosporium, Curvularia, Penicillium, Rhizopus, Periconia, Pythium, Trichothecium, etc. prevailed on the roots and root zone of corn. Heterotrophic species of fungi belonging to the genera Alternaria, Bipolaris, Exserohilum and Fusarium were found more often on the leaves. It should be noted that the frequency of occurrence of fungi Alternaria spp. depended on the phenological phase of corn plants. The pathogen Exserohilum turcicum was identified on the leaves of corn of the lower tier. The causative agent Bipolaris sorokiniana mainly infected the roots and the basal part of the corn stalk during the formation phase of 5–6 leaves.

During ontogenesis, 11 species of fungi from the genus Fusarium were found on corn crops in the Voronezh region: F. culmorum, F. gibbosum, F. graminearum, F. heterosporum, F. fujikuroi, F. incarnatum, F. oxysporum, F. poae, F. roseum, F. sporotrichioides, F. solani. Among them, two species, F. heterosporum and F. oxysporum, were noted with high frequency. Similar types of pathogens on corn have been identified by foreign scientists (Ali et al., 2005; Eller et al., 2008). During many years of research, V.G. Ivashchenko and colleagues identified 15 species of fungi of fusarium etiology on corn crops in Russia (Ivashchenko, 2012).

It has been shown that pathogenic and phytotoxic activity in fungi varies significantly between Fusarium species and within the same species. The greatest danger to corn is represented by fusarium fungi of the following species: F. sporotrichioides, F. graminearum, F. culmorum, F. fujikuroi, F. oxysporum, F. heterosporum, which have a high intensity of phytotoxic activity associated with the ability to synthesize and accumulate dangerous toxins in plant tissues. The results of similar studies were previously obtained by us when detecting pathogenic and phytotoxic activity of fungi from the genus Fusarium isolated from affected wheat plants. Isolates of F. culmorum, F. graminearum, F. heterosporum, F. oxysporum isolated from wheat had a wide intraspecific diversity according to these characteristics. Among them, as well as on corn, isolates of pathogens with different levels of pathogenic and phytotoxic activity were found (Zhemchuzhina et al., 2021).

The nature of the effect of FCF strains of fungi F. graminearum, F. heterosporum, F. fujikuroi, F. solani and F. redolens on barley seedlings is characterized by high phytotoxicity, and F. avenaceum, F. poae, by weak phytotoxicity. Of all the listed species, F. sporotrichioides and F. sambucinum isolates turned out to be the most pathogenic and phytotoxic on barley. In F. culmorum and F. oxysporum species, in contrast to those isolated from corn, the frequency distribution of all categories of pathogenicity and phytotoxicity was approximately the same (Kolomiets et al., 2018).

Thus, as a result of mycological studies conducted on affected maize samples from the Voronezh region, the State Collection of phytopathogenic microorganisms of ARRIP was replenished with 55 strains of fungi belonging to seven types of pathogens from the genus Fusarium. The selected strains of phytopathogens, stable in morphological and cultural characteristics, characterized by pathogenicity and phytotoxicity, have been stored for long-term storage using lyophilization and cryopreservation methods.

## Conflict of interest

The authors declare no conflict of interest.
